# Genetics and genetic counseling in psychiatry: Results from an opinion survey of professionals and users

**DOI:** 10.1002/mgg3.830

**Published:** 2019-06-29

**Authors:** Lourdes Martorell, Annabel Sanfeliu, Ana Blázquez, Elia Lojo, Maria José Cortés, Joan de Pablo, Elisabet Vilella

**Affiliations:** ^1^ Hospital Universitari Institut Pere Mata, IISPV, Universitat Rovira i Virgili Reus Spain; ^2^ Centro de Investigación Biomédica en Red en Salud Mental (CIBERSAM) Madrid Spain; ^3^ Department of Child and Adolescent Psychiatry and Psychology Clinic Institute of Neurosciences, Hospital Clínic de Barcelona Barcelona Spain; ^4^ Germans Trias i Pujol Hospital. UAB Badalona Spain

**Keywords:** genetic counseling, genetics, patients, professionals, psychiatry

## Abstract

**Background:**

The heritability of several psychiatric disorders is high, and specific at‐risk variants have been identified. Therefore, genetic counseling and genetic testing can be prescribed to some psychiatric patients, but these services are not standardized for most of the population. The aims of the study were to gather opinions from mental health professionals and users regarding (a) the genetics of psychiatric disorders and (b) the usefulness of a genetic counseling unit in psychiatry.

**Methods:**

The survey was conducted in the province of Tarragona (Spain), and we analyzed 152 valid questionnaires from professionals and 959 from users.

**Results:**

Sixty‐one percent of professionals strongly believed that psychiatric disorders have a genetic basis, and 59% rated a genetic counseling unit in psychiatry as very or extremely useful. However, only a few professionals reported that patients asked them about the genetics of their diseases (12%) or the possibility of transmitting the disease to offspring (19%). Forty‐seven percent of users strongly believed that psychiatric disorders have a genetic basis, 30% responded that they talked with their families about the genetics of their diseases, and 43% were worried about transmitting the disease to offspring; however, only 14% reported that their psychiatrist had talked to them about this topic. Remarkably, 80% of users would consider a genetic counseling unit very or extremely useful.

**Conclusions:**

The present study showed that mental health professionals were more aware of the genetic basis of psychiatric disorders than users, and both considered the implementation of a genetic counseling service very useful.

## 1 INTRODUCTION

Currently, a substantial amount of evidence confirms the importance of genetic contributions to mental illnesses. Heritability estimates for almost all psychiatric disorders are in the range of 0.30–0.80; 0.35 for major depressive disorder (MDD) (Otte et al., 2016), 0.75 for bipolar disorder (BD) (Sullivan, Daly, & O’Donovan, 2012), 0.40 for obsessive compulsive disorder (OCD) (Pauls, Abramovitch, Rauch, & Geller, 2014), 0.30–0.80 for anorexia nervosa (AN) (Shih & Woodside, 2016), 0.70–0.80 for attention‐deficit hyperactivity disorder (ADHD) (Voeller, 2004), and 0.80 for both autism spectrum disorder (ASD) (Colvert et al., 2015) and schizophrenia (SCH) (Kahn et al., 2015). The rest of the variability is known to be caused by environmental factors, such as stressful life situations, brain damage, cannabis abuse, and childhood maltreatment (Schmitt, Malchow, Hasan, & Falkai, 2014), and interactions between genes and the environment through epigenetic mechanisms. In fact, the presence of a psychiatric illness in a patient's biological relatives is a strong risk factor for many psychiatric disorders. Moreover, some genetic factors are shared between distinct psychiatric diagnoses, suggesting a common genetic background (Smoller et al., 2013; Torres, Barbosa, & Maciel, 2016).

Both single nucleotide polymorphisms (SNPs) and copy number variants (CNVs) are implicated in the development of psychiatric disorders. Genome‐wide association studies have identified hundreds of SNPs with very small effects that confer a risk for a wide range of psychiatric disorders (Cross‐Disorder Group of the Psychiatric Genomics Consortium, Ripke, Neale, Faraone, & Purcell, 2013). Regarding CNVs, compelling data suggest that a small number of recurrent deletions and duplications increase the risk of several neurodevelopmental disorders, including ASD, SCH, BD, seizure disorder, and intellectual disability (ID) (Jiang et al., 2014; Kirov et al., 2014). Interestingly, approximately 65% of the recurrent CNVs are de novo variants (Rees, Moskvina, Owen, O’Donovan, & Kirov, 2011), indicating that they are not inherited. Of these, 22q11.2del is the most prevalent CNV associated with SCH and ASD. Recently, both 22q11.2 deletion and duplication have been associated with an increased risk of any psychiatric disorder and with a highly increased risk of ID in a Danish nationwide, register‐based study (Hoeffding et al., 2017). In addition to SNPs and CNVs, rare coding variants (RCVs), which have an allele frequency <1:1,000 and affect a single or a few nucleotides, have been demonstrated to exert considerable effects on the risk for psychiatric disorders (O’Donovan & Owen, 2016).

Genetic counseling is the process of advising individuals and families affected by or at risk of genetic disorders to help them understand and adapt to the medical, psychological, and familial implications of genetic contributions to disease (Resta et al., 2006), and it is neither synonymous with nor dependent on genetic testing (Moldovan, Pintea, & Austin, 2017). Genetic testing is a part of genetic counseling when a genetic test is available to detect a DNA alteration, such as the 22q11.2 CNV; however, this practice is currently very limited in mental health settings (Moldovan et al., 2017). A large component of genetic counseling is informing affected subjects about the risk of developing or transmitting a disease and the associated consequences when subjects plan to have children. However, part of genetic counseling in psychiatry aims to help affected individuals adapt to mental illness, understand the etiology of their disease, and increase empowerment and positive self‐identity (Costain & Bassett, 2012; Hippman et al., 2016; Inglis, Koehn, Mcgillivray, Stewart, & Austin, 2015).

Knowledge of the genetics of psychiatric diseases may allow genetic counseling for symptomatic and nonsymptomatic patients; however, genetic counseling is currently not broadly offered to psychiatric patients and families in most developed countries (Moldovan et al., 2017). The genetic contribution to psychiatric disorders is not more complex or less significant than the genetic contribution to other complex diseases for which genetic counseling is traditionally offered. For example, at least in Europe and specifically in Spain, the public health system offers genetic testing and genetic counseling for primary dyslipidemia (Wiegman et al., 2015), a complex disease estimated to affect 2%–3% of the adult population. However, in Spain and many other countries, these services are not offered for severe psychiatric diseases that affect a similar range of people and impose a serious burden on patients and society, at least in a generalized manner. The main reason for not offering genetic counseling regarding psychiatric disorders in Catalonia is the lack of specific regulation. Several other factors may be involved in this situation. For example, only approximately 20% of psychiatrists consider themselves competent to provide genetic information to patients (Finn et al., 2005; Venugopal, Ranjith, & Issac, 2000), although most psychiatrists feel that they should provide such information (Jenkins & Arribas‐Ayllon, 2016). Psychiatrists and other mental health professionals report the need to improve their knowledge and skills in psychiatric genetics and even report that a genetic counseling service that accounts for the complexity of psychiatric conditions (uncertainty of psychiatric diagnoses, patient engagement, and a limited capacity to understand) is necessary and effective (Jenkins & Arribas‐Ayllon, 2016; Moldovan et al., 2017). On the other hand, no valid, high‐certainty diagnostic psychiatric genetic tests are currently available for common complex psychiatric disorders. In this sense, psychiatric genetic counseling and psychiatric testing are considered emerging disciplines in Europe, and efforts to develop a framework to facilitate the implementation of both disciplines into routine clinical care are currently underway (https://www.cost.eu/actions/CA17130).

In this context, we developed two questionnaires, one directed at psychiatric health professionals and another directed at patients and relatives, to survey their opinions about the genetics of psychiatric disorders and to explore the possible demand for a genetic counseling service focused on psychiatric disorders.

## 2 SUBJECTS AND METHODS

### 2.1 Sample and setting

The survey was conducted in the province of Tarragona in southern Catalonia, Spain. This area had a population of 800,962 in 2014 and a total area of 6,308 km^2^, with a relative population density of 127 habitants per km^2^ and an economic output of €29,400 GDP per capita. Our institution provides all public mental health care in this region through a network of community and hospitalization centers, with patients ranging from infants to elderly people; further details can be found in Gaviria et al. (2015). The study was approved by the Clinical Research Committee of the Hospital Universitari Institut Pere Mata. Participation in the survey was voluntary, and the participation procedure was designed in such a way that it was completely anonymous and therefore did not require the request for written informed consent and approval by the Ethical Committee. No compensation was offered for participation.

#### 2.1.1 Professional sample

We sent the survey by institutional e‐mail to all 723 professionals, including psychiatrists, general practitioners, neurologists, psychologists, mental health nurses, clinical assistants, social care workers, social educators, occupational therapists, and other health professionals. The e‐mail contained a link to an online questionnaire prepared with the Cardiff Teleform^R^ System (Electric Paper GmbH, Lachen, Switzerland). The completed questionnaire was automatically sent to a server and incorporated into the study database in an anonymous form.

#### 2.1.2 User sample (Patients and Relatives)

In 2015, 16,372 people had at least one clinical checkup in our institution: 11,024 in adult mental health centers, 4,651 in child and adolescent mental health centers, and 697 in the center for people with both psychiatric disorder and intellectual disability. Paper questionnaires, which were prepared to be automatically read with the Cardiff Teleform^R^ System, were left in specific trays of the waiting rooms of seven adult and seven child and adolescent mental health service centers and in the mental health with intellectual disability community center, and were distributed throughout the territory. Near the trays, flyers and posters explaining the nature and aim of the survey were displayed. Appropriate and identifiable ballot boxes were available to deposit the anonymous questionnaires. The research team refilled the trays weekly with paper questionnaires for the entire recruitment period (5 months), and a total of 1,500 questionnaires were distributed.

### 2.2 Procedures

#### 2.2.1 Instruments

Two survey instruments were designed by academic and clinical experts in psychiatry and psychiatric genetics addressing three domains of interest: sociodemographic data, knowledge about the genetics of psychiatric diseases, and the utility of a psychiatric specialized genetic counseling unit. Two identical questions were addressed to both professionals and users. The specific questions were “To what extent do you think psychiatric disorders have a genetic basis?” and “To what extent do you think that a genetic counseling service would be useful?” We also asked professionals about their opinions on the genetic basis of specific common psychiatric disorders and intellectual disability, the level of education in the genetics of psychiatric disorders, and whether they discuss genetic questions about psychiatric disorders with users. We also asked users whether they were worried about transmitting a disease to children and whether they talk about genetic concerns with their relatives or mental health professionals. The instruments were written in both Catalan and Spanish, and the English versions were produced only to be shown here. Two members of the research team that conducted the survey translated the Catalan/Spanish questionnaires into English and two independent researchers from our group, but not members of the survey research team back translated the English questionnaires into Catalan/Spanish. No discrepancies were observed between the two versions.

The professional questionnaire (Figure S1) consisted of eight questions regarding genetics in mental health, and the respondents indicated their agreement with each item on a 10‐point Likert scale ranging from 0 (never or not at all) to 10 (almost always or extremely). Moreover, age, sex, work setting, and job title were collected, together with the respondents’ reports of the diagnoses of patients who they most frequently helped.

The user questionnaire (Figure S2) consisted of five questions rated on a 10‐point Likert scale similar to that for the professional questionnaire. In addition to age and sex, participants were asked whether they were psychiatric patients or relatives of psychiatric patients, their education level, and the number of affected relatives and their diagnoses.

### 2.3 Data analysis

Descriptive statistics were obtained from the total sample and from the subgroups of participants and were calculated using percentages, means, and standard deviations. Statistical tests were used not to test hypothesis, but to explore the possible effect of age, sex, setting, career field, or education in the responses. Chi‐square tests were used to compare categorical variables with Yale's continuity correction if appropriate, and Mann–Whitney *U* tests and Kruskal–Wallis tests were used for numeric variables. ANOVA was used to compare item responses between different age groups. The normality of the distributions of continuous variables was explored using Kolmogorov–Smirnov normality tests.

Questions with answer options between 1 and 10 were categorized to facilitate interpretation of the results. To recode the answers, the following categorization was performed: 0 (never or not at all), 1–3 (seldom or slightly), 4–6 (sometimes or moderately), 7–9 (often or very), and 10 (almost always or extremely). Ratings ≥7 were considered “positive answers,” and ratings ≤3 were considered “negative answers”; ratings between 4 and 6 were considered neither positive nor negative. Analyses completed with the original codification system ranging from 0 to 10 and with the recoded data showed similar results.

The professional sample was stratified by sex (male/female), ranked age (20–35, 36–45, 45–55, 56–65, and >65 years), career field (children and adolescents, adults, intellectual disability, and geriatrics), and profession (psychiatrist, psychologist, mental health nurse, social care worker, social educator, occupational therapist, other medical specialties, administrative staff, and others). The user sample was stratified by sex (male/female), ranked age (≤18, 19–35, 36–45, 46–65, and >65 years), who completed the survey (patient/relative) and educational level (primary, secondary, and upper secondary vs postsecondary school, bachelor, or equivalent).

All statistical analyses were performed using IBM SPSS Statistics for Windows, Version 23.0 (IBM Corp., Armonk, NY). Graphs to represent the results were created with Prism, Version 5 (GraphPad, La Jolla, CA).

## 3 RESULTS

**Figure 1:**
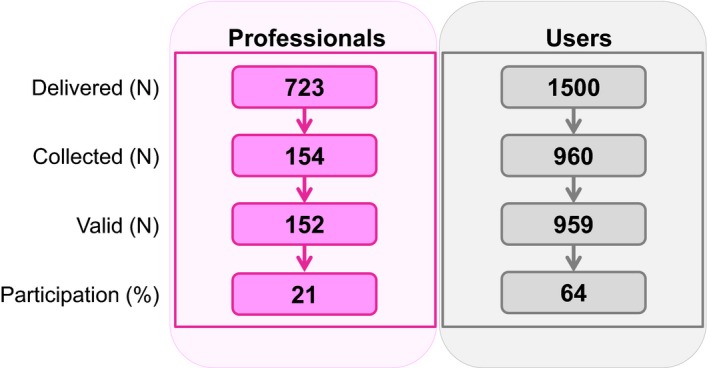
Flow diagram of participants in the survey

We obtained 152 and 959 valid questionnaires from professionals and users, respectively, which represented 21% of the invited professionals and 64% of the questionnaires delivered to users (Figure 1). Interestingly, most of the respondents, including 78% of professionals and 67% of users, were women. Table 1 shows the sociodemographic features of the survey participants. Participants ≤45 years old represented 76% of the professionals and 53% of the users. Psychiatrists, psychologists, and mental health nurses accounted for 64% of the professional participants according to job title. Regarding the users, 63% were patients and 37% were relatives of patients. Among the patients, 5% were younger than 18 years, 7% were older than 65 years, and 88% were between 18 and 55 years old. Sixty‐nine percent of the users who participated in the survey had either a primary or secondary education, which is usually completed at the age of 16 in Spain, while 22% had obtained a university education.

**Table 1:** Characteristics of the participants in the survey

Professionals	*N*	(%)	Users (63% patients, 37% relatives)	*N*	(%)
*Sex*			*Sex*		
Male	34	(22.4)	Male	307	(32.7)
Female	118	(77.6)	Female	631	(67.3)
*Age, y*			*Age, y*		
			<18	51	(5.3)
20–35	53	(34.9)	18–35	188	(19.6)
36–45	63	(41.4)	36–45	265	(27.6)
46–55	29	(19.1)	46–55	262	(27.3)
56–65	7	(4.6)	56–65	130	(13.6)
			>65	63	(6.6)
*Setting*			*Setting*		
Child–Adolescent	25	(16.4)	Child–Adolescent	166	(17.3)
Adult	81	(53.3)	Adults	504	(52.6)
Intellectual disability	31	(20.4)	Intellectual disability	14	(1.4)
Psychogeriatrics	15	(9.9)	Mixed, child and adult	275	(28.7)
*Career field*			*Education*		
Psychiatrist	36	(23.7)	Primary school, unfinished	101	(10.5)
Psychologist	35	(23.0)	Primary school	246	(25.7)
Mental health nurse	26	(17.1)	Secondary school, unfinished	75	(7.8)
Social worker	16	(10.5)	Secondary school	239	(24.9)
Occupational therapist	2	(1.3)	Upper secondary school	87	(9.1)
Social educator	6	(4.0)	Bachelor or equivalent	211	(22.0)
Other	31	(20.4)			

### 3.1 Questions addressed to both professionals and users

**Figure 2:**
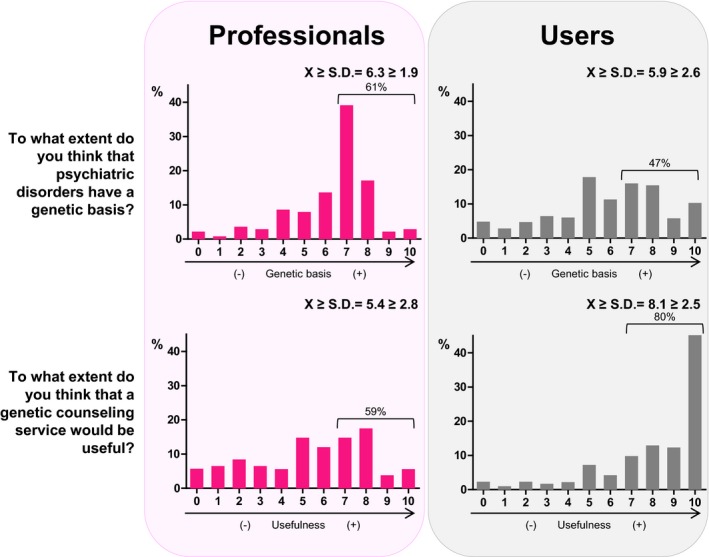
Distributions of the responses obtained from professionals and users to the two identical questions presented to both groups. Participant ratings ranged from 0 (never or not at all) to 10 (almost always or extremely), which are presented on the X‐axis. The Y‐axis shows the percentages of participants who provided each rating

Two identical questions were addressed to both professionals and users. Regarding the question, “To what extent do you think psychiatric disorders have a genetic basis?,” the average rating of professionals was 6.3 ± 1.9 (Figure 2). Although 61% of them believed that psychiatric disorders have a genetic basis (rated ≥ 7), 9.1% thought that psychiatric disorders do not have or rarely have a genetic basis (rated ≤ 3). The extent to which professionals felt that a genetic basis for psychiatric disorders exists was not significantly associated with job title or clinical setting. Higher ratings were obtained with increasing age, but no significant differences were observed (*p* = 0.234). Female professionals rated this question higher compared to male professionals (*p* = 0.023). For the same question regarding the genetics of psychiatric disorders, the average rating for users was 5.9 ± 2.6 (Figure 2), which is similar to the rating of 6.3 ± 2.0 observed among professionals (*p* = 0.106). Almost half of the users, 47%, believed that psychiatric disorders are often or almost always related to genetics (rated ≥ 7). At the opposite pole, 5% of users thought that psychiatric disorders do not have a genetic basis at all (rated 0). Similar to professionals, the user group aged 36–55 years showed higher ratings for this question compared to the other age groups (*p* < 0.001). Additionally, patients and relatives with the highest level of education (university studies) provided the highest ratings for this question (*p* = 0.038).

Regarding the second question addressed to both professionals and users, “To what extent do you think that a genetic counseling service would be useful?,” 59% of professionals believed that having a genetic counseling unit would be very or extremely useful (rated ≥ 7), while only 16% thought that it would seldom or never be useful (rated ≤ 3) (Figure 2). Interestingly, 80% of users thought that a genetic counseling unit would be very or extremely useful. Notably, 45% of users rated this question at the maximum value (10), while only 2% thought that having a genetic counseling service would be useless (Figure 2). Users rated the usefulness of a genetic counseling service question higher than professionals, 8.1 ± 2.5 versus 5.4 ± 2.8, respectively (*p* < 0.001). The youngest professionals, those aged 20–35 years, rated this question higher than the oldest professionals (*p* = 0.048), with higher ratings associated with females (*p* < 0.001).

### 3.2 Questions addressed to professionals

**Figure 3:**
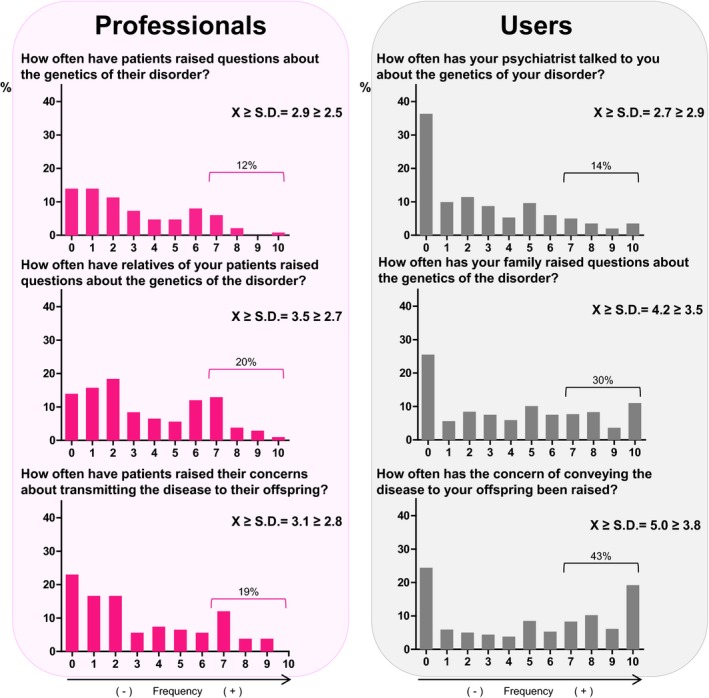
Distributions of the responses obtained from professionals and users to different questions in the questionnaires. Participant ratings ranged from 0 (never or not at all) to 10 (almost always or extremely), which are presented on the X‐axis. The Y‐axis shows the percentages of participants who provided each rating

**Figure 4:**
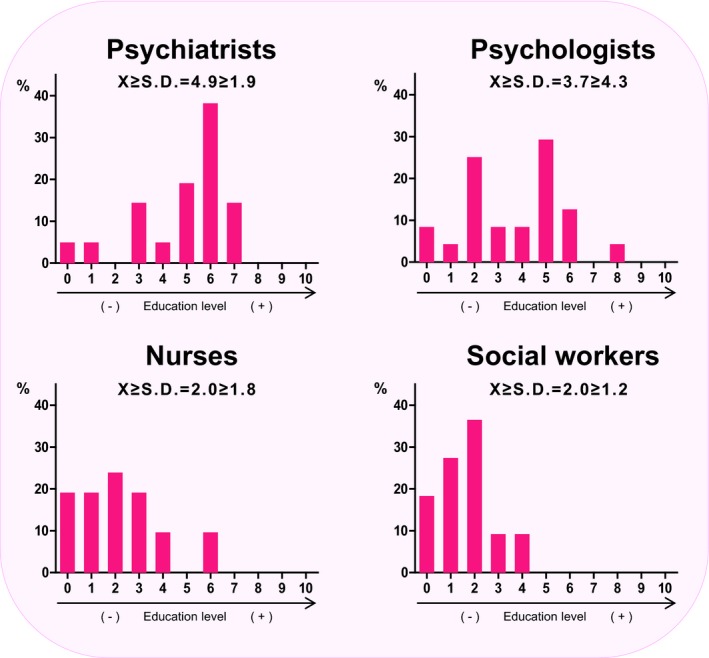
Self‐reported level of education regarding the genetic basis of psychiatric disorders by distinct professional profiles. Participant ratings ranged from 0 (never or not at all) to 10 (almost always or extremely), which are presented on the X‐axis. The Y‐axis shows the percentage of participants who provided each rating

Professionals were asked about issues focusing on the knowledge and worries that users discuss with them regarding the genetic basis of their illnesses. Approximately 12% of professionals provided a rating of ≥7 (often or almost always) for the question, “How often have patients raised questions about the genetics of their disorder?” On a similar question regarding relatives instead of patients, the percentage of professionals who gave a rating ≥7 was 20% (Figure 3). Female professionals tended to rate the frequency of patients asking them about the genetic basis of their disease higher (*p* = 0.039). Likewise, higher ratings were provided by professionals working in adult mental health centers (*p* < 0.001) and from psychologists (*p* = 0.016) compared to other groups in the same category. Regarding the concern of transmitting a disease to offspring, 19% of professionals stated that patients often or almost always (rated ≥ 7) asked them about this issue. On the other hand, 59% of the professionals believed that they had a low level (rated ≤ 3) of education on the genetics of psychiatric disorders (Figure 4). In fact, only 8% reported having a high level of education on this topic (rated ≥ 7). The average rating of all professionals for this question was 3.2 ± 2.4. The stratified analysis revealed that psychiatrists rated this question higher compared to other professionals, with a mean rating of 4.9 ± 1.9; the other mean scores were 3.7 ± 4.3 for psychologists, 2.0 ± 1.8 for nurses, and 2.0 ± 1.2 for social workers (*p* = 0.007) (Figure 4). Regarding the possibility of sending patients to a genetic counseling unit, 41% of professionals indicated that they would often or almost always send patients to such a unit (rated ≥ 7) compared to 27% who reported that they would seldom or never send patients to such a unit (rated ≤ 3).

### 3.3 Questions addressed to users

Regarding the question, “How often has your psychiatrist talked to you about the genetics of your disorder?,” 14% reported often or almost always (rated ≥ 7). Remarkably, 36% of users responded that their psychiatrists had never (rated 0) talked about this issue, and 30% reported that their psychiatrists rarely addressed this issue (rated between 1 and 3) (Figure 3). Therefore, 66% of users have seldom or never talked about the genetics of their illnesses with their psychiatrists (rated ≤ 3). Regarding the question, “How often has your family raised questions about the genetics of the disorder?,” 30% reported often or almost always (rated ≥ 7), but 25% of the users had never (rated 0) talked about this issue. Differences were observed based on age: users aged 18–35 responded that psychiatrists talked with them about genetics more frequently compared to older subjects (*p* = 0.017). In addition, women gave higher ratings compared to men (*p* = 0.001). Regarding the concern of transmitting an illness to offspring, 43% of users reported that they have often or almost always been concerned about this issue, and 19% were almost always concerned (rated 10). Notably, however, 39% of users have rarely or never been concerned about this topic (rated ≤ 3). The diagnoses reported by users to be present in their family members are shown in Table 2.

**Table 2:** Diagnoses indicated by users, patients (*N *= 604), and relatives (*N *= 335), to be present in their family members

Diagnosis	Patients		Relatives	
	*N*	(%)	*N*	(%)
Schizophrenia	108	(18)	54	(16)
Bipolar disorder	120	(20)	57	(17)
Major depressive disorder	156	(26)	57	(17)
Autism spectrum disorder	18	(3)	55	(16)
Obsessive compulsive disorder	81	(13)	33	(10)
Attention deficit and hyperactivity disorder	66	(11)	89	(26)
Anxiety disorder	269	(45)	104	(30)
Behavior disorder	62	(10)	56	(16)
Personality disorder	107	(18)	40	(12)
Adaptive disorder	47	(8)	26	(8)
Addictive disorder	55	(9)	25	(7)
Intellectual disability	25	(4)	48	(14)

## 4 DISCUSSION

The present study found that in our setting, a considerable number of professionals and users believe that psychiatric disorders have a genetic basis; however, users of mental health centers do not often comment on or discuss with psychiatrists the genetic basis of the psychiatric disorders with which they have been diagnosed. Similar data were obtained when asking both psychiatrists and users. Remarkably, a considerable number of users reported that their psychiatrist never talked to them about the genetics of their illness. This finding contrasts with the findings that nearly half of users have often or almost always been concerned with transmitting a disease to their offspring, and that a considerable number of users, almost one‐third, reported that questions about the genetics of the disease have arisen often or almost always within their families. Thus, our results indicate that this topic is not addressed in psychiatrists’ consultations despite patients’ concerns. Genetic factors are relevant to the etiology of most psychiatric disorders. It the information age, patients can be expected to want to know the causes of their illnesses. Therefore, in daily clinical practice, patients can be expected to ask about and discuss the etiology of their diseases with physicians, especially given that most of the professionals in our setting are aware of the genetic basis of psychiatric disorders. However, the results of the present survey were not consistent with this expectation and allowed us to speculate about possible explanations. On the one hand, knowledge of psychiatric genetics has increased substantially over the last decade, and these recent advances, along with the complexity of the genetic basis of psychiatric disorders, may lead professionals to avoid talking in depth with patients, particularly when a considerable amount of time may be necessary to appropriately address this subject. On the other hand, other factors may render this topic difficult to discuss, including the presence of clinical and genetic heterogeneity, comorbidity with some neurological conditions in a considerable number of cases, and the large number of genes and types of genetic variants involved. Finally, environmental factors play an important role in the etiology of psychiatric disorders, and large differences in genetic and environmental factors between psychiatric disorders have been reported, thus complicating the determination of their etiological roles (Pettersson et al., 2019). Obviously, the lack of a genetic counseling unit to which patients can be referred is also a disadvantage when discussing the genetic and environmental contributors to the etiology of illnesses with patients. Experienced genetic counselors agree on the necessity of equipping health‐care professionals with current knowledge of psychiatric genetics/genomics and, moreover, effectively communicating these aspects with patients, especially when genetic testing is performed (Hoang, Cytrynbaum, & Scherer, 2018). Therefore, genetic counselors must be trained in the genetic basis of psychiatric disorders. In this sense, extensive insight can be gained from the experiences of the world's first genetic counseling service in psychiatry in Vancouver, with professionals who have treated more than 500 families and demonstrated the benefits of this service in terms of patient empowerment and self‐efficacy after attending genetic counseling (Inglis et al., 2015). This service has recently reported interest among users in a prenatal context in knowing the likelihood of their children developing an illness present in the family (Borle, Morris, Inglis, & Austin, 2018) and how they can address this issue with prenatal genetic counseling (Inglis, Morris, & Austin, 2017). The authors showed that this context also serves as an opportunity to engage patients and to help them feel empowered. Ideally, this training should be generalized to other mental health providers, especially psychiatrists, who are in contact with users, either patients or relatives. Accordingly, the International Society of Psychiatric Genetics recently reported that informed genetic counseling in psychiatry is in greater demand than ever and recommends that genetic education should become an integral part of psychiatric training (Nurnberger et al., 2018).

Analytically and clinically valid genetic tests for neurodevelopmental disorders, including Fragile X syndrome, Down's syndrome, and neurodegenerative diseases such as Huntington's disease are available. Therefore, genetic counseling can be offered to screen at‐risk individuals before the onset of symptoms, before clinical diagnosis, or to establish the diagnosis after symptoms have appeared. However, an estimated 30 million Europeans are related to patients affected by genetic conditions that may have been underrecognized by the health system. This finding contravenes the European Union's aim to create safe, efficient, patient‐centered, and sustainable health‐care systems (McAllister, Moldovan, Paneque, & Skirton, 2016). In fact, the global number of genetic counselors has been estimated to be small, although genetic counseling is considered a growing area (Abacan et al., 2019; Cordier, Lambert, Voelckel, Hosterey‐Ugander, & Skirton, 2012; Ormond et al., 2018). The situation is much worse for psychiatric disorders. Genetic counseling services do not routinely provide assistance to patients with psychiatric disorders, although we currently know that several recurrent CNVs can confer a substantial risk for ASD, SCH, epilepsy, developmental delays, and congenital malformations (Kirov et al., 2014). Although CNVs have been implicated in these disorders, CNV genetic analysis is usually recommended only for children (Jeste & Geschwind, 2014; Schaefer, Mendelsohn, & Professional Practice and Guidelines Committee, 2013). Regarding ASD, a recent study has explored access to genetic counseling in six ASD family associations in Catalonia, a geographical area that includes our province. The authors identified extensive underutilization of this service, with only 30% of all families receiving a genetic service and only 13% of patients undergoing genetic screening as recommended (Codina‐Solà, Pérez‐Jurado, Cuscó, & Serra‐Juhé, 2017). Indeed, at least in Catalonia, adult patients affected by psychiatric disorders with onset in adolescence or adulthood, such as schizophrenia, are not genetically screened, although these recurrent risk CNVs have been estimated to be present in 2.5% of schizophrenia patients (Kirov et al., 2014; Rees et al., 2011). In addition to the interest that patients may have in accessing genetic tests, especially patients with schizophrenia, genetic counseling may play an important role within the framework of psychiatric/psychotherapeutic treatment because genetic testing has psychological, ethical, and clinical implications, as indicated in the “Genetic Testing Statement” published by the Genetic Testing Working Group from the International Society of Psychiatric Genetics (https://ispg.net/genetic-testing-statement/).

Finally, female professionals and users are more aware and concerned about the genetics of psychiatric disorders compared to males. Female professionals also reported a higher likelihood of discussing genetics with patients compared to male professionals. Among the reasons that may explain these gender differences, women may have stronger association with reproduction; therefore, patients feel that questions about inheritance may be more appropriate for women versus men. Another reason may be that women professionals may be more empathetic and thus relatable. Therefore, the gender perspective must be considered, and this issue should be included in future training programs and addressed appropriately.

The strength of this study is the large number of mental health users who participated. However, several limitations should be mentioned, including the relatively small number of professionals who participated in the study considering the number of professionals working in our institution. Additionally, the study was conducted in a province with only one public mental health provider with a research group devoted to studying the genetic basis of psychiatric disorders, which may have influenced the results of the survey. Although our data were collected from a single province in Spain, these results are likely generalizable to most regions in developed countries.

In summary, the benefits of genetic counseling even without genetic testing have been demonstrated. In addition, evidence supports the contributions of SNPs, CNVs, and RCVs to the development of psychiatric disorders. In some cases, the genetic component of a psychiatric disease is highly heritable within a family; however, in a significant number of cases, the genetic variant has occurred in the germ line and manifest as a de novo mutation. Therefore, people suffering from a psychiatric disorder with high heritability, namely, SCH or ASD, should visit a genetic counseling unit, at least during the reproductive period. Likewise, families with psychiatric illness aggregation should also be evaluated. Finally, in addition to these two approaches focusing on high heritability and high aggregability, the relatively high number of CNVs that occur as de novo mutations justifies screening for this type of mutation, at least in all severe psychiatric patients, especially when present with comorbid conditions. Performing more tests and uploading more data in specific databases will increase the usefulness of the genetic screening and counseling. In this sense, uploading genetic information as well as phenotypical and environmental data that would translate from the clinical to research settings would be helpful.

The results of the present study showed that both professionals and users are aware of the genetic basis of psychiatric disorders, and both believe that access to a genetic counseling service would be very useful. Notably, although they did not report asking their psychiatrists about the genetics of psychiatric disorders, most patients were concerned about these issues and are interested in psychiatric genetic counseling.

## CONFLICT OF INTEREST

The authors declare no conflict of interest.
