# Supplementary figures and images for: Genetics and genetic counseling in psychiatry: Results from an opinion survey of professionals and users
Source: Mol Genet Genomic Med. 2019 Jun 29;7(8):e830. doi: 10.1002/mgg3.830 (PMC6687663; doi:10.1002/mgg3.830)

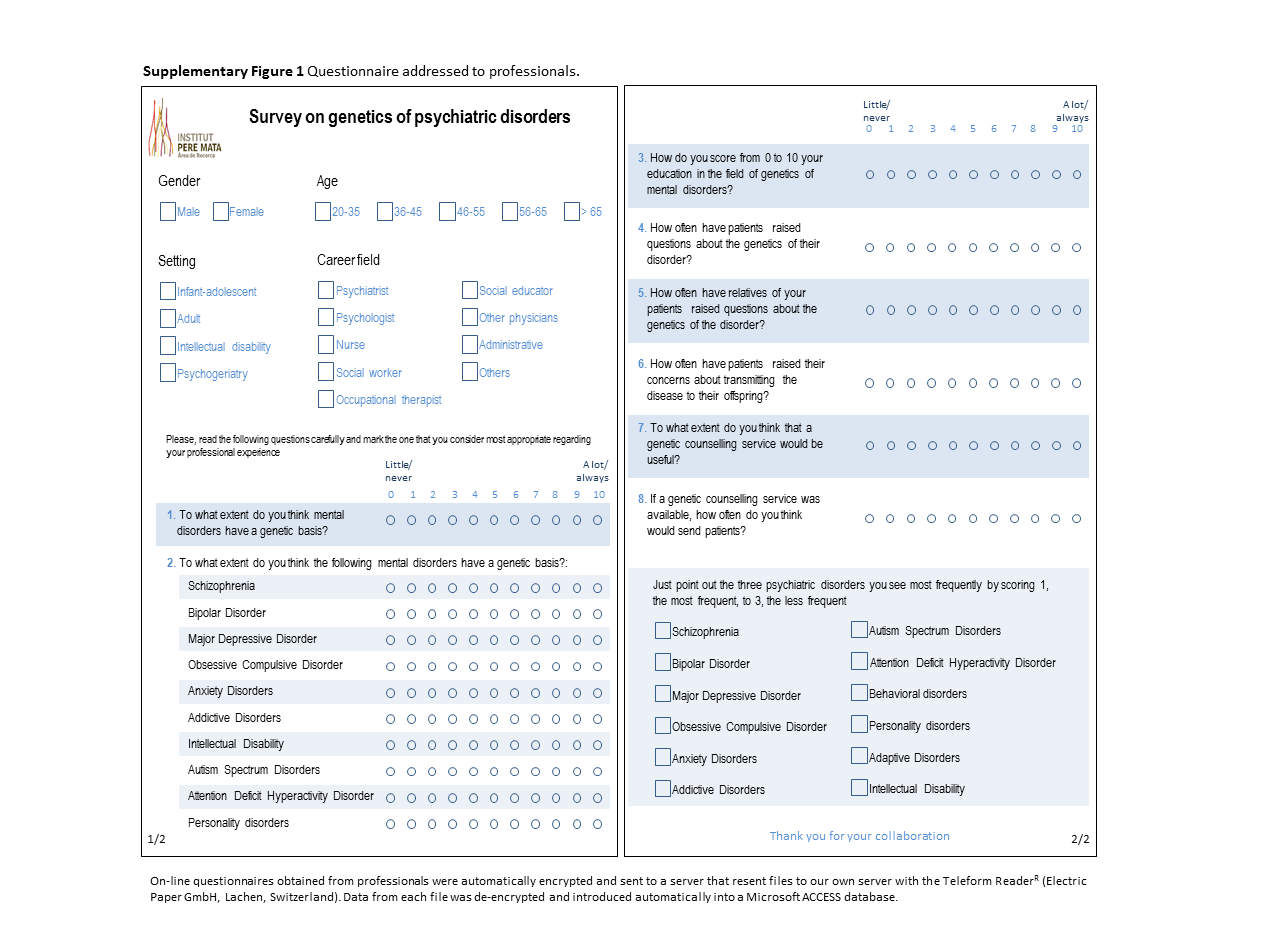

Supplement: Supplementary file 1 [file MGG3-7-e830-s001.tif]

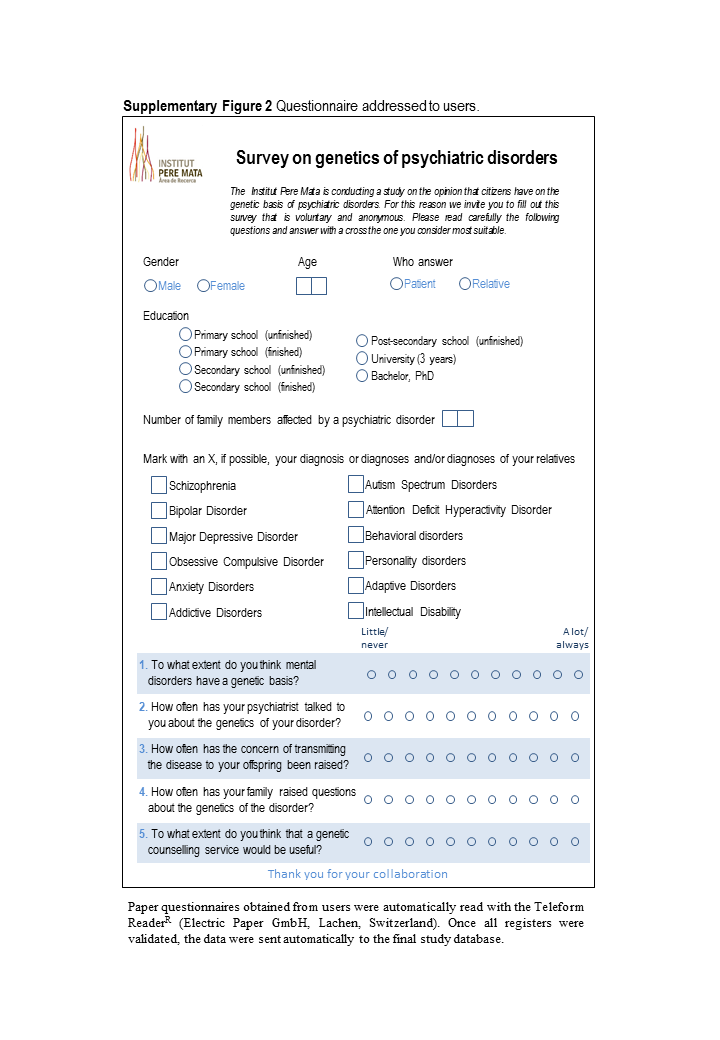

Supplement: Supplementary file 2 [file MGG3-7-e830-s002.tif]
